# Atomistic Origin of RTN-like Centers Created and Annihilated by RRAM Write Processes

**DOI:** 10.1021/acs.nanolett.5c06450

**Published:** 2026-04-27

**Authors:** Paul Solomon, Manasa Kaniselvan, Hiroyuki Miyazoe, Babar Khan, Takashi Ando, Mathieu Luisier

**Affiliations:** † 3261 IBM T.J. Watson Research Center, 1101 Kitchawan Rd, Yorktown Heights, New York 10598, United States; ‡ Integrated Systems Laboratory, Department of Information Technology and Electrical Engineering, ETH Zürich, CH-8092 Zürich, Switzerland

**Keywords:** valence change memory, ReRAM, resistive switching devices, memristor, kinetic Monte Carlo

## Abstract

We examine in detail random-telegraph-like noise (RTN) generated in resistive random-access memory (ReRAM) structures as a consequence of applying programming pulses. We find that a single programming pulse can induce highly correlated RTN noise patterns, in terms of frequency and amplitude, which are replaced by a new pattern on the next programming pulse. A weaker ‘repair’ pulse is ineffective at reducing the RTN frequency or amplitude. Remarkably, similar patterns are seen in our atomistic simulations of ReRAM structures, which reveal occasional bursts of semiperiodic and constant amplitude current pulses. These can be traced to reversible back and forth oscillation of a set of point defects modulating the conductance of an electro-formed channel. This is the first report of sustained conductance oscillations caused by reversible configuration changes in VCM cells.

## Graphical abstract

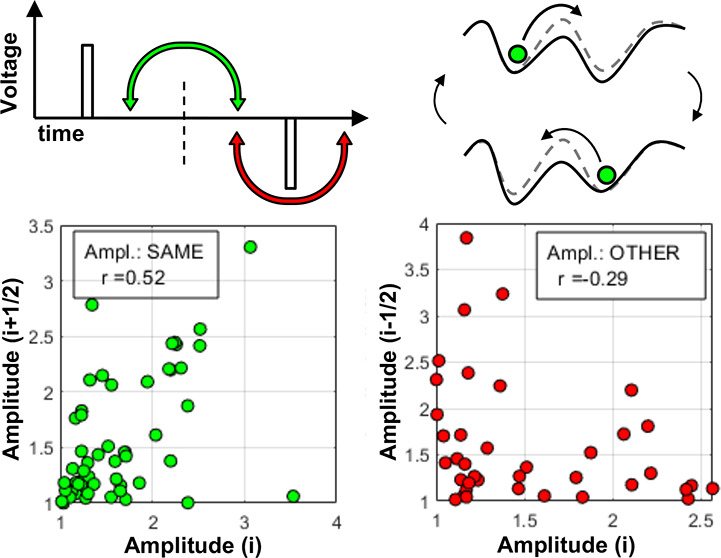

Resistive Random Access Memory (ReRAM) is an attractive candidate as building blocks of extremely dense digital and analog memory applications, as well as of in-memory compute schemes based on crossbar arrays of memristors.^1,2^ The physics of their operation involves the formation of nanometer sized conductive filaments^3^ within a dielectric material, created through a soft dielectric breakdown (forming) process. The dielectric of the ReRAM is typically made of a metal-oxide, MO_*x*_, (hafnia, zirconia, alumina etc.). Subsequently, the device can be programmed into higher or lower resistance states through application of write pulses, usually of opposite polarity. The programming mechanism is attributed to the formation and transport of oxygen vacancies.^2,4^ This process leaves behind nanoscale filaments of oxygen vacancies in the bulk MO_*x*_, which change the valence of surrounding M atoms and render them conductive, hence its categorization as a ‘Valence Change Memory’ (VCM).^5^ Comprehensive reviews of the microscopic breakdown processes are given in Padovani et al.^6^ and, specifically relating to VCM, in Dittman et al.^4^

A serious problem limiting widespread use of VCM technology is the stochastic nature of the switching processes, accompanied by large read and write noise commonly associated with Random Telegraph Noise (RTN).^2,7–12^ For example, Gong et al.^9^ quantified noise observed during incremental conductance changes representing weight updates for neural network training, and found a trade-off between symmetrized switching - needed for multistate compute in memory - and signal-to-noise ratio. The origin of these RTN-like transitions remains a subject of research.

Classical RTN, as in most electronic devices, is assumed to result from filling and emptying electronic traps, either by directly modulating the conductance of a narrow filament through trap-assisted tunneling^8,10,13^ or remotely modulating the channel conductance through the field effect.^11,14^ RTN-like oscillations of small amplitude (<1 μA) in ReRAM can be readily explained by electron trapping-detrapping processes. Electronic RTN is characterized by stationary statistics, yet experimental patterns can often be more complex when several traps participate, appearing as a combination of two or more RTN sequences with different time and voltage characteristics.^10,14^ In addition, reconfiguration of the channel can cause large jumps in the current, modifying the RTN pattern.^12,13,15,16^ Rao et al.^11^ investigated this phenomenon in depth in TaO_2_ devices, and determined that the write process induces a large number of RTN-like defects created as a result of incomplete secondary filaments and function as electron traps. These defects were imaged by a conductive AFM probe and could be effectively healed with a series of lower-voltage write pulses that suppressed these filaments.

However, ReRAMs have also exhibited RTN-like transitions occurring at larger amplitudes,^13,17^ and in temporal bursts.^10,17^ Such changes can be triggered by programming (SET and RESET) operations. Subsequent ‘noise’ in the measured conductance may result from different types of postprogramming relaxation processes.^15,17^ Unlike conventional electronic devices, the operating principles of ReRAMs includes ionic movement. These larger-amplitude transitions have thus been theorized to occur via the displacements of vacancies within the current path of the filament, thus directly modulating the current flow.^4,13,15^

Dittmann et al.^4^ propose that the range of observed RTN falls into one of three categories: (1) steady state fluctuations (i.e., charge modulation), (2) trap assisted tunneling, or (3) configurational changes in the filament due to random jumps of oxygen vacancies. Similar classification types were also introduced by Tanaka et al.^12^ The ionic movements involved in the operation of ReRAMs may enable transitions following this third mechanism. Recently, the ability of single atom movements to cause such large current fluctuations in VCM devices has been theoretically shown through *ab initio* Quantum Transport simulations.^18^ We theorize here that it lies at the origin of the observed burst-like transitions in the noise measurements of ReRAM.

In this letter we first present pulse induced RTN observations and show that, in our samples, a single programming pulse can induce a burst of up to hundreds of RTN transitions where the RTN statistics are (1) correlated within the pulse period but also (2) decorrelated from pulse to pulse. Unlike Rao et al., we were not able to significantly reduce the programming-induced noise by applying reduced level pulses. We then propose that the transitions we observed result from atomic rearrangement events, and support our theory with observations of this phenomenon from Kinetic Monte Carlo simulations.

Our device under test consists of an ReRAM stack (Figure 1(a)) in series with an integrated 10KΩ resistor, as in Figure 1(b). The stack comprises of a TiN bottom electrode, a 5 nm-thick HfO_2_ layer, and a TiN top electrode. We first switch the sample with long series of up-and-down pulses to bring it near its ‘symmetry point’.^9^ Note that this differs from most previous work, where the ReRAM cell is typically switched between high and low resistance states. The symmetry point characterizes the state of the ReRAM at which applying positive/negative programming pulses of equal magnitude causes its conductance to oscillate around a fixed value, representing a ‘steady state’. This condition can be visualized in Figure S1. This point is desirable for physical investigations of switching because the device is poised for switching with equal probability in both directions. It is also more appropriate to emulate the weight updates in neural network applications.^19^

**Figure 1:**
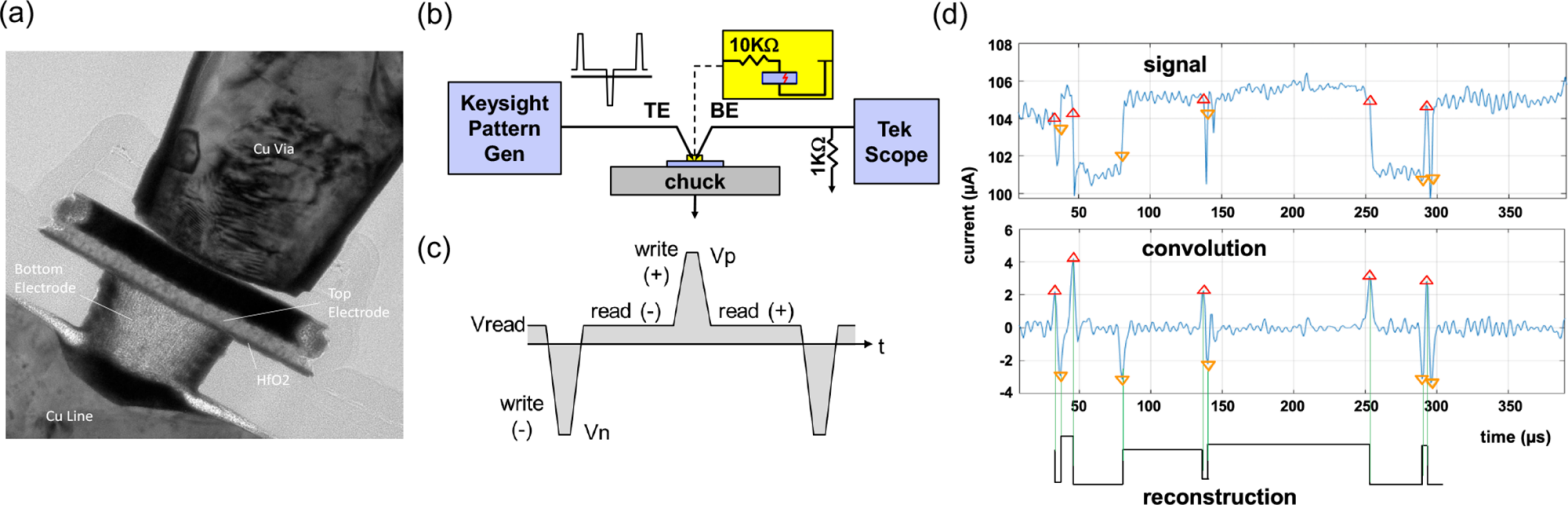
Device and noise measurement setup, showing (a) a cross-sectional TEM image of the ReRAM stack considered here and (b) our experimental setup for high-speed and high-timing-precision write and read operations including an integrated 10 KΩ resistor. (c) Details of the applied waveforms. (d) RTN data extraction showing an example of signal current (μA) vs time (ms). The signal (top pane) is convolved with the kernel [−1 −1 −1 1 1 1] and its inverse. Positive and negative peaks are indicted with up and down triangles in the center pane. Paired up/down transitions are then designated as valid RTN transitions. An example of a reconstructed signal is shown in the bottom pane.

The current is then monitored over a 20s period which includes the high speed programming pulses and read intervals (Figure 1(c)), at a fast-sampling rate (typically 100 kHz to 1 MHz). The latter is fast enough to capture most RTN events. The RTN data was extracted from the transient trace using convolution and peak extraction, as pictured in Figure 1(d). Discrimination is achieved using a minimum peak height and accepting as valid transitions those occurring as ± or ∓ pairs. Typically, thousands to tens of thousands of RTN-like pulses may be recorded in a 20 s trace (Table S1). Typical evolution of conductance, conductance modulation, read and write noise are shown in the Supporting Information, Figure S2. The excess read noise can be traced to RTN-like transitions (Figure S3), where a variety of relaxation-type processes are observed (Figure S4).

Patterns of read noise, on application of up and down programming pulses, can vary widely from sample to sample or even in the same sample, reflecting the stochastic nature of the filament evolution. The thousands of up/down programming pulses bring the sample to a constant current, its symmetry point, as indicated in Figure S2 (a, e). Figure S5 shows a wide range of behaviors on the same sample, under pulse conditions listed in Table S1. We derive our main results from this sample. Figure 2(a-b) displays the current fluctuating around the symmetry point, with most of the fluctuations due to the modulation caused by the programming pulses. The RTN, seen in the insets, often appears in bursts bounded by the programming pulses. The statistics of these transitions are far from Poissonian, as pictured in Figure 2(c) where a strong excess in large burst size is evidenced. This implies that the transitions originate from correlated processes. The period of the RTN pulses (Figure 2(d)) is not strongly influenced by the programming period and is in the fractional millisecond range. Similar behavior is reported in Figure S6 with much shorter pulses, and pulse period, in a different experimental arrangement. While the statistics of pulse number per read period is strongly non-Poissonian, the statistics of the first RTN transition following a programming pulse is exponential in nature, with a time-constant in the fractional millisecond range (Figure S7). This strongly evidence the action of the programming pulses (both + ve and −ve) in initiating RTN.

**Figure 2:**
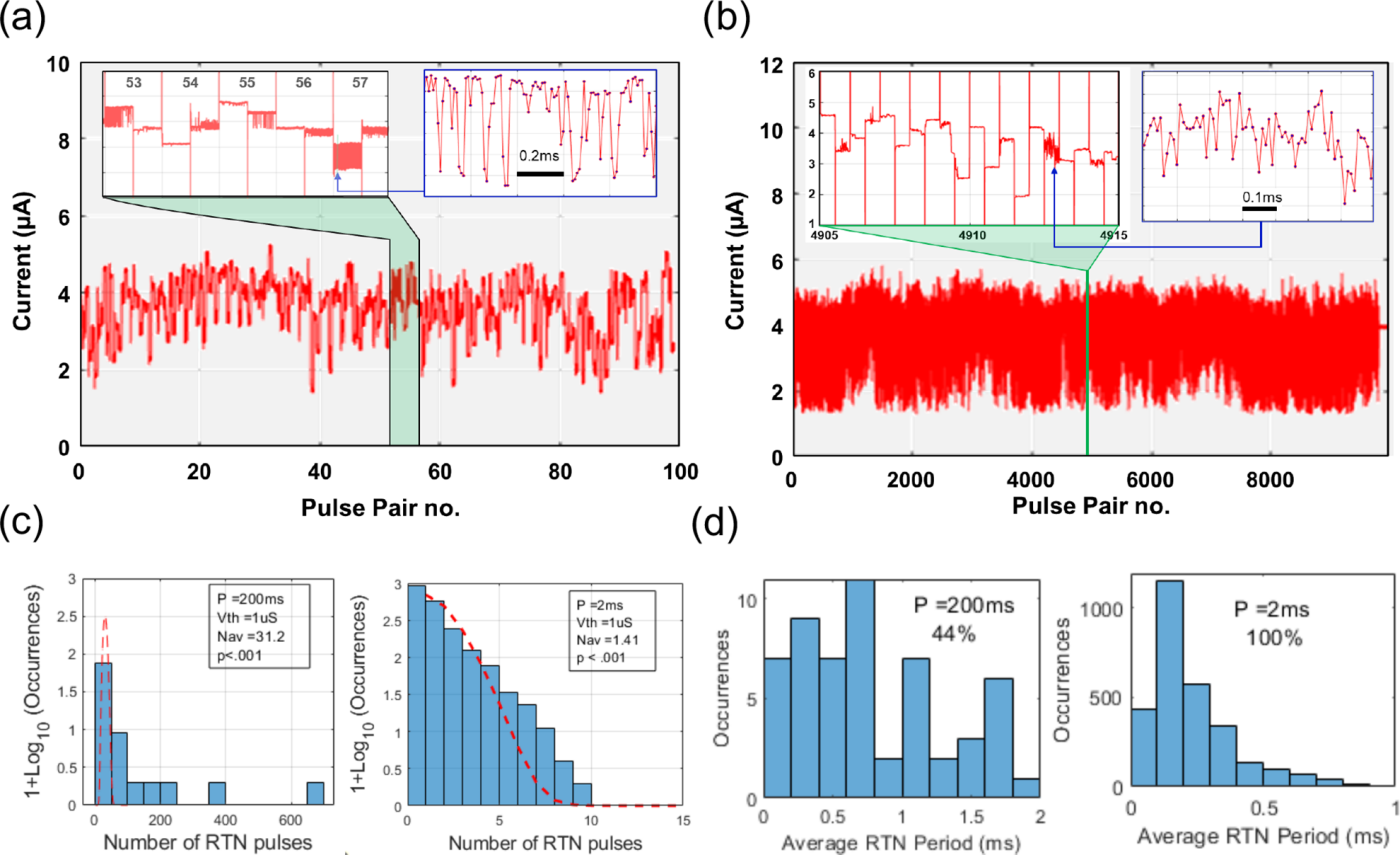
Write-induced RTN-like pulses on two different time scales with (a) 200 ms and (b) 2 ms pulse pair period. The pulse pair number is used in lieu of a time scale. The insets show a magnified time segment, together with the 1 μs pulses that were eliminated from the main figures for clarity. The sampling points in the doubly magnified insets are 10 μs apart. The full programming parameters are listed in Table S1 of the Supporting Information. (c) Histograms of the logarithm of the number of transitions compared to the shape of a log Poisson distribution (red dashed line) with the same average. (d) Histograms of the RTN pulse period for two different programming periods and the percentage of events within the horizontal scale of the figure. The p-values in (c) indicate the probability of obtaining test results at least as extreme as the result observed, assuming the null (Poissonian) hypothesis is true. Thus, a small p-value rejects the Poissonian hypothesis.

In some cases, the average RTN amplitude was found to be strongly correlated with average modulation amplitude (Figure S8). However, for the present samples, the correlation, at the individual programming pulse level is weak, even though, in general there is tracking between the average modulation and RTN amplitudes. The lack of 1–1 correlation is brought out sharply in Figure S9, where examples are presented of ‘strong modulation but no RTN’ and ‘strong RTN but no modulation’.

We examine the case of ‘strong RTN in the absence of modulation’ in more detail in Figure 3. The write pulses are small (± 25 mV) and can thus be ignored. Here we see Markovian-like steps in the average conductance (Figure 3(a)), as has been observed by others.^4,13,17^ However, at smaller time scales (Figure 3(b)) we see much faster activity patterns superimposed on the average. This behavior belies the explanation of other studies^13,17^ that the change in average conductance is simply a change in the conductance of a single channel brought about by the transition of a single vacancy, rather it is characteristic of the transition between complex states of high entropy.

**Figure 3:**
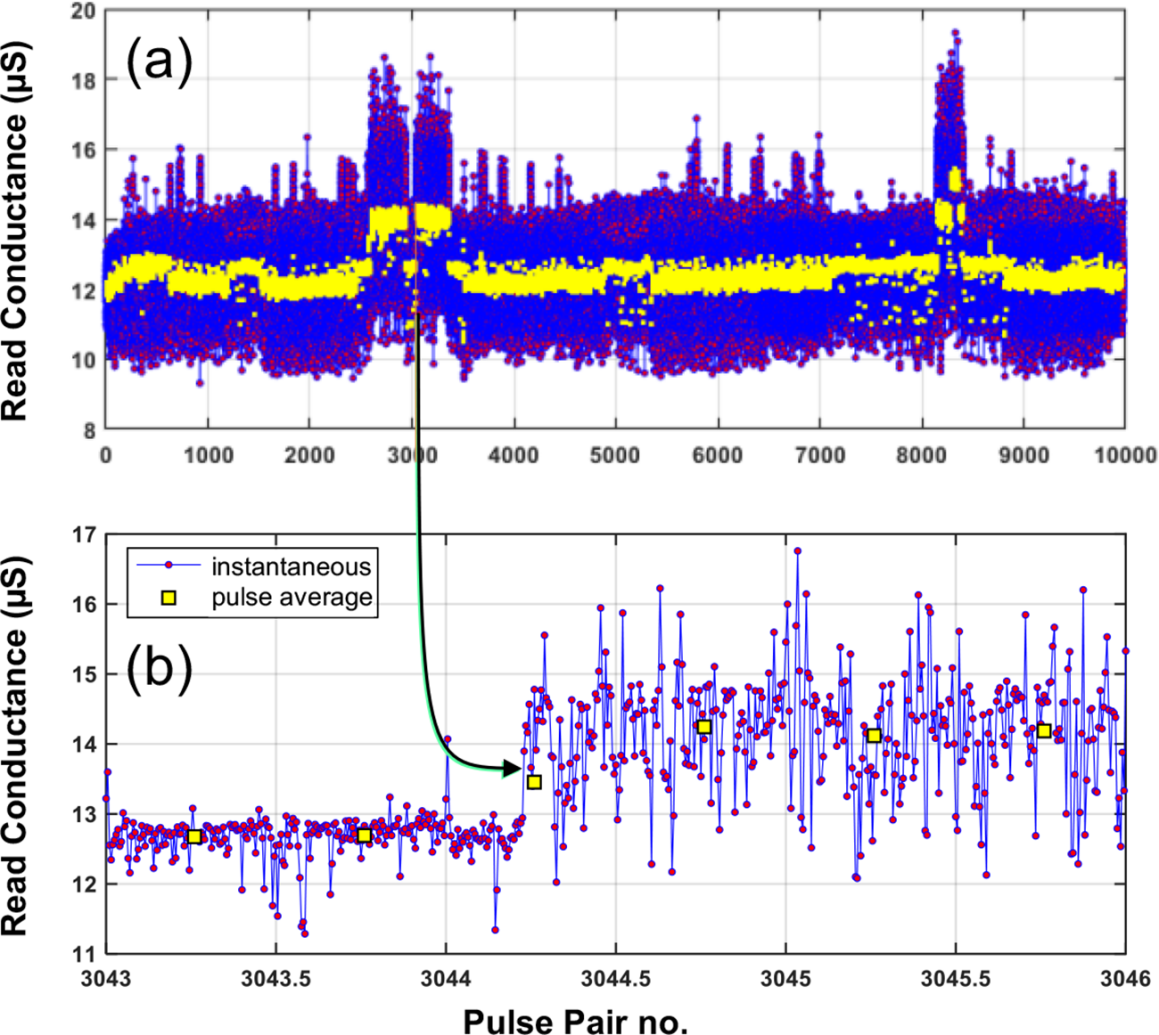
Sample showing extensive RTN with minimal write pulses. (a) Average current per programming pulse (yellow) superimposed on the instantaneous value. Same as (a), but on an expanded time scale (2 ms per pulse pair). Apparent slow state transitions correspond to the superposition of many, much faster, microtransitions with much larger amplitude dispersion. The sampling period is 10 μs.

In Figure 4(a-c), we investigated the RTN statistics over the 20s trace, using both (1) statistical correlations between pulses within the same programming cycle and (2) pulses from adjacent cycles, as pictured in Figure 4(a). The quantities compared in Figure 4 are (b) the mean pulse amplitude per $\frac{1}{2}$ period, and (c) mean pulse number. It can be observed that both the mean amplitude (Figure 4(b)) and pulse number (Figure 4(c)) are more strongly correlated within a segment than across segments. The peak/valley time ratio (duty factor, Figure 4(d)), however, was found to be weakly correlated, or uncorrelated, even within the same segment.

**Figure 4:**
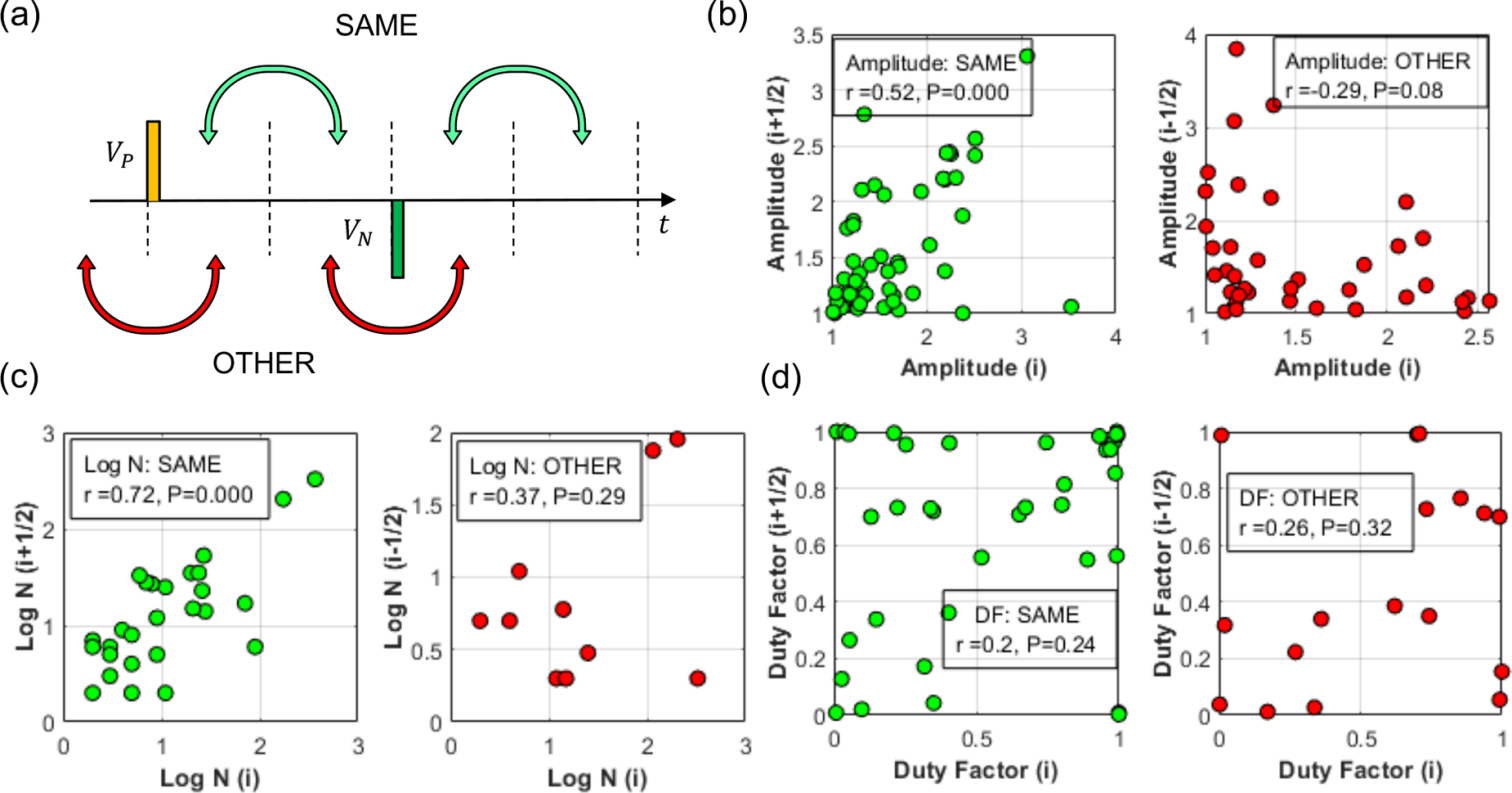
Correlation plots by $\frac{1}{4}$ period, where RTN amplitudes over adjacent $\frac{1}{4}$ periods are compared either within (SAME) or across (OTHER) $\frac{1}{2}$ period programming pulses. (a) Schematic view of the programming pulses V_*p*_ and V_*n*_. (b–d) Correlations of RTN mean amplitude, RTN mean number, and duty factor with the annotation giving the correlation coefficient and the statistical p-factor. Any value above p = 0.05 implies the hypothesis of correlation is rejected. The pulse pair period was 200 ms.

Rao et al.^11^ (and similarly, Yamada et. al^12^) demonstrated that RTN could be greatly suppressed by applying reduced amplitude programming pulses, or ‘healing’ pulses. However, their experiments involved an iterative repair procedure with multiple pulses. This is practical for a program-once application such as inference, but is unsuited to applications involving frequent state updates, where the latency penalty would be prohibitive. We therefore tested whether a similar effect can be achieved using a single-pulse repair process. We applied a healing pulse midway between the two, opposite polarity, programming pulses (see Figure S10 and analyzed the statistics for healing pulses ranging from −0.75 to +0.75 V. In these experiments we did not see any significant healing effect, but it is evident that the intersegment correlation, strong at first, decreases for the larger healing pulses i.e. the healing pulses act just like additional programming pulses generating their own, uncorrelated, RTN.

Next, we adopt a Kinetic Monte Carlo (KMC) model^5^ to study the plausibility of ionic transition-induced RTN. KMC-based simulations have shown success in capturing the kinetic phenomena underlying resistive switching in ReRAM stacks.^4,10,13,15^ Specifically, they can simulate the atomistic evolution of point defects in the presence of a changing energy landscape influenced by external fields, from which microscopic phenomena such as the formation of conductive filaments naturally arise. Our KMC technique can simulate these structures free of artifacts of a numeric grid, thus preserving the richness of the energy landscape.^5^

The simulated RRAM devices are constructed of a material stack consisting of the same binary-oxide used for the fabricated devices (amorphous HfO_2_), as well as a thin layer of Ti which acts as an oxidizable reservoir (Figure 5(a)). Activation energies for vacancy/ion generation, diffusion, and recombination events are initially computed with Nudged Elastic Band simulations, and are continuously modified by local potential differences across neighboring atomic sites. Bond-resolved currents are computed with a 3D resistive-graph model parametrized with direct- and trap-assisted tunneling- (TAT) terms. The parameters of this internal current solver are calibrated against quantum transport calculations across an HfO_2_ structure. The power collected at every point is multiplied by a factor (α) modeling the fraction dissipated as heat in the oxide,^20^ and input to the heat transfer equation solved on the same graph to simulate Joule heating within the oxide. The heat equation is discretized onto a finer, regular time scale, which allows for the simulation of transient heat buildup and thermal runaway effects. The contacts are modeled as heat sinks (set to 300 K). Implementation details can be found in ref 5. The simulated domain sizes (2.5 × 2.5 × 5 nm oxide dimensions) are considerably smaller than the switching region of the fabricated devices. However, resistive switching devices are operational at such dimensions,^21^ and the constriction points of conductive filaments in HfO_2_ have been imaged at the nanometer scale,^3^ indicating that the underlying physics is captured in such small domains. We note that in these aggressively scaled domain sizes, individual atomic movements can lead to large variations in current.^18^

**Figure 5:**
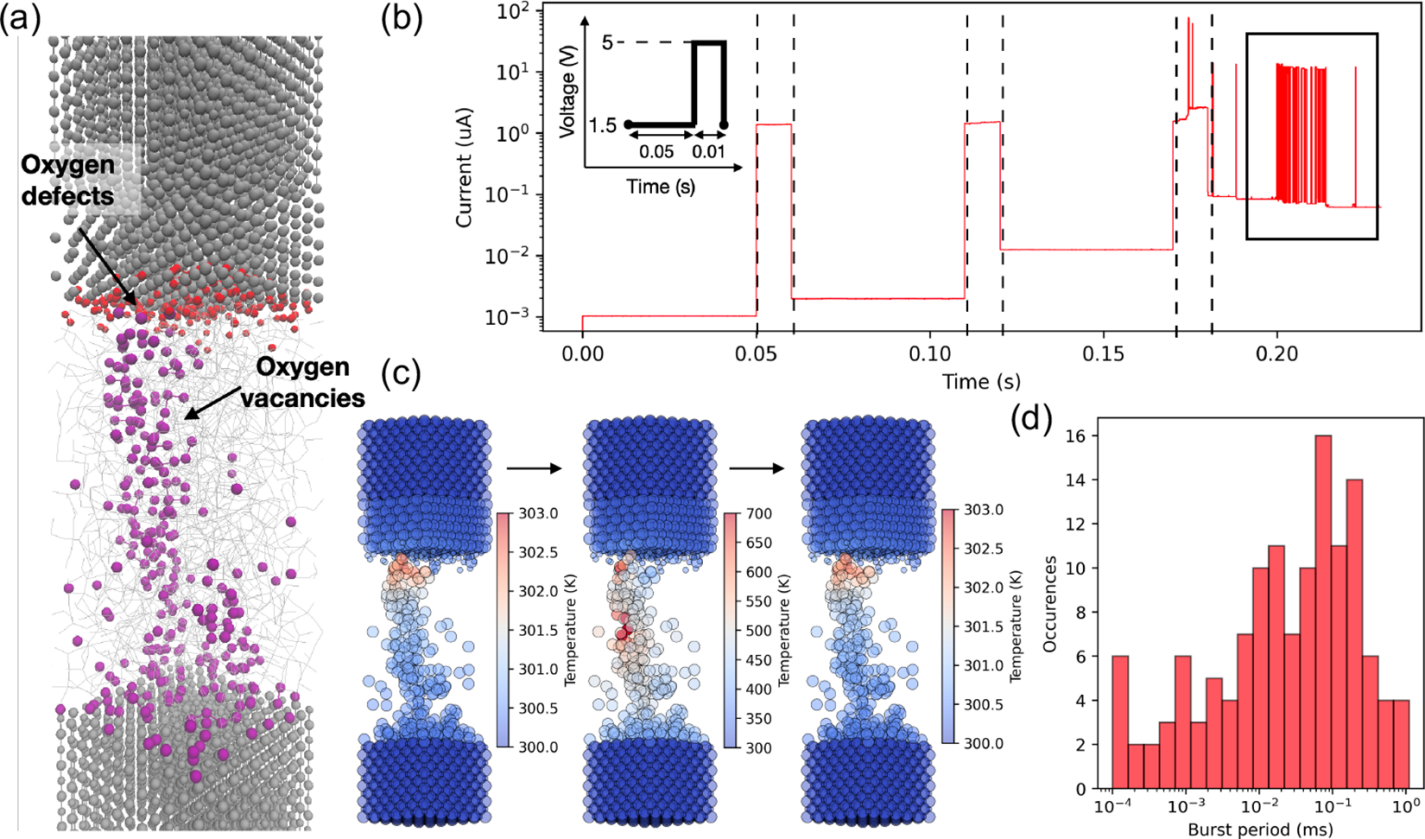
(a) Atomistic description of the device used as input into a KMC simulation tool. Colored spheres indicate oxygen defects, which have left their lattice positions to oxidize the interface, and oxygen vacancies, which conduct current. (b) Current as a function of three consecutive applied pulses on the device in (a) following the biasing scheme in the inset. (c) Difference in temperature profiles taken from three consecutive snapshots in the black box within (b), showing the change in heat as the filament is locally disrupted and recreated by the motion of a single oxygen atom. We omit the oxide atoms for visual clarity. (d) Distribution of noise periods extracted from the transitions within the black box in (b).

To test if the oscillations observed experimentally can be induced in simulations, we apply the pulsed voltage scheme detailed in the inset of Figure 5(b) to a structure initially in a moderately high resistance state, and track the changes in current (Figure 5(b)). Due to the absence of electronic effects in our simulations, most of the measured currents are relatively constant. However, on occasion we see rapid fluctuations in current – one of such cases is highlighted with the black box in Figure 5(b). Similarly to experimental observations, these oscillations are stochastic (e.g., dependent on the random numbers generated during the weighted Monte Carlo selection process), and cannot be exactly predicted, other than that we observe they appear with higher likelihood in incomplete filaments under higher electric fields, where more complex energy landscapes are produced.

By examining the change in atomic positions within simulated domain, the oscillations in computed current can be traced to the movement of single oxygen vacancies or, alternatively, through the substitution of nearby oxygen atoms into the void corresponding to a vacancy, a defect migration process. Figure 5(c) shows magnified snapshots of this process, this time with each atom colored by its local instantaneous temperature profiles. We use these temperatures as a proxy to visualize where current flow is ‘bottle-necked’ in the structure. Changes in the localization of heat indicate changes in the connectivity of conductive sites. These connectivity changes result from individual atomic migration events, each of which locally modifies the potential landscape, generated heat, and changes the magnitude and distribution of electrical current flow through the switching layer. The high- and low- currents across the oscillation region specifically correspond to a filamentary pathway being repeatedly completed/disrupted by intermittent movement of an oxygen atom. As this pathway is delicate, the current - and thus the instantaneous increase in generated heat – is bottle-necked through the narrow constriction formed in the neighborhood of the oxygen vacancy left behind. As the same one- to few-atoms participate in this transient process, this mechanism is consistent with the experimentally measured correlation in atomic movements within a single measurement period. Supplementary Video 1 shows an animation of this process happening, connecting the atomic displacement events to abrupt changes in current flow. The typical period of the simulated RTN transitions within this burst is in the fractional millisecond range (with a peak at ∼0.1 ms, Figure 5(d))), which correlates well with the experimentally measured periods in Figure 2(d) ((∼0.5–0.1 ms)).

Experimentally, the RTN was observed under read conditions where the voltage applied (0.1 V) was too low to cause programming events. On the other hand, larger programming pulses caused modulation of the device’s conductance. The following characteristics were observed: **(1)** RTN statistics are reset by preceding programming pulses. **(2)** The amplitude of the RTN pulses is large, similar to the modulation amplitude, yet there is no 1–1 relationship between the two. Sometimes correlation is observed but at other times the two seem to be independent of each other. **(3)** Programming pulses are shown to initiate and terminate RTN bursts. Bursts of up to hundreds of RTN pulses were observed. **(4)** RTN amplitude and pulse number were correlated within a programming interval but not across intervals. On the other hand, the peak/valley ratio (duty factor) appears to be uncorrelated, even within an interval. It appears that the programming pulses scramble the RTN, resetting the clock. Smaller-amplitude ‘repair’ pulses do not quench the RTN, but when they are large enough, they simply reset the clock, decorrelating the RTN.

Our results are consistent with RTN due to atomistic rather than electronic transitions. While the electronic RTN noted in Rao et al.^11^ originates from isolated oxygen vacancies which can exist in a charged state, our RTN signals likely come from oxygen vacancies within the filamentary pathway, as proposed by refs 8, 12, 13 . In case of a change in charge state of a vacancy outside of the main filament, the modulation of the current path is attenuated and the magnitude of current which can flow through this mechanism is thus limited, producing low-magnitude fluctuations. In contrast, current flow through a vacancy at a filament bottleneck location causes a combination of local heating and change in the local potential which can incite atomic movements to occur. This coupling of atomic movements and electronic current flow together can lead to much larger changes in current. Strong evidence for the atomistic explanation is the burst-like nature of the RTN. RTN caused by electronic traps should have fairly stationary statistics, contrary to our observations. Unlike electronic RTN, where peak/valley ratio is constant for a given bias,^22^ no correlation was observed in our case.

A similar burst-like behavior is seen in our atomistic simulations, where atomic migration and relaxation within the filament result in a narrow window favorable to RTN transitions. The multilevel, multitime-scale behavior revealed in Figure 3 shows that, in the absence of programming, very complex behaviors can develop over time. The picture that emerges is that of a very dynamic environment where the Markovian steps^13,17^ are the average of many, much faster, blocking events. The RTN bursts can be both initiated and terminated by programming pulses. Yet, in our case the termination does not result in reduced RTN noise since initiation is more likely to dominate.

Perhaps this analogue can explain the difference: Electronic RTN has a virtually infinite reservoir of electrons to draw from, resulting in a statistically stationary RTN pattern, at a given bias. In contrast, the number of atoms which determine the current flow through narrow, filamentary constrictions, is far smaller and finite, and any large oscillation of the position of a current-gating defect will quickly be damped out. Indeed, it seems improbable that after a violent programming event an active defect such as a vacancy may be precariously balanced between two iso-energetic sites such that it can oscillate back and forth hundreds of times, yet our simulations mimic such activity.

## Supplementary Material





## Notes

Further experimental device characterization (PDF)
Short video showing the kinetics of the simulation plotted in Figure 5 (MP4)
