# Supplementary material for: Atomistic Origin of RTN-like Centers Created and Annihilated by RRAM Write Processes
Source: Nano Lett. 2026 Apr 27;26(17):5695–701. doi: 10.1021/acs.nanolett.5c06450 (PMC13154351; doi:10.1021/acs.nanolett.5c06450)
Supplement: Supplementary file 1 [file nl5c06450_si_001.pdf]

Atomistic Origin of RTN-like centers created and Annihilated by RRAM write Processes - Supplementary Materials

Paul Solomon,[*,†] Manasa Kaniselvan,[‡] Hiroyuki Miyazoe,[†] Babar Khan,[†] Takashi Ando,[†] and Mathieu Luisier[*,‡]

†*IBM T.J. Watson Research Center, 1101 Kitchawan Rd, Yorktown Heights, N.Y. 10598.*

‡*Integrated Systems Laboratory, Department of Information Technology and Electrical Engineering, ETH Zürich, CH-8092 Zürich, Switzerland*

E-mail: solomonp@us.ibm.com; mluisier@iis.ee.ethz.ch

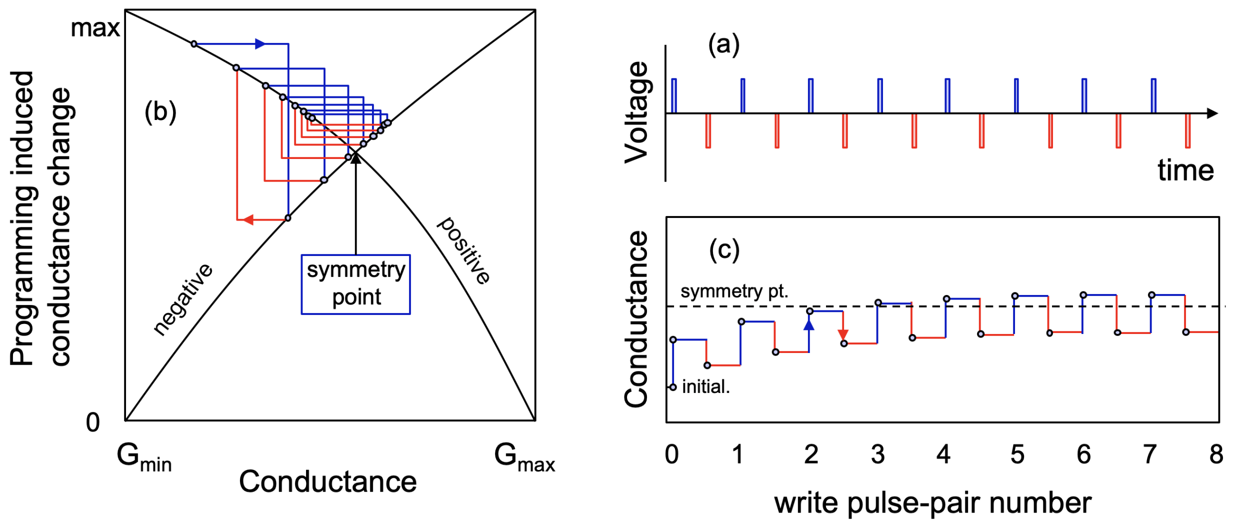

Figure S1: The ReRAM element can be programmed by positive or negative pulses which (a) increase or decrease its conductance respectively (b) according to non-linear programming curves. (c) Repeated +/- programming pulses bring the device close to its symmetry point (indicated in (b)), where the positive and negative programming increments are equal, with the conductance oscillating about the symmetry point value. The blue and red lines in (b) and (c) show trajectory of conductance after positive and negative programming pulses.

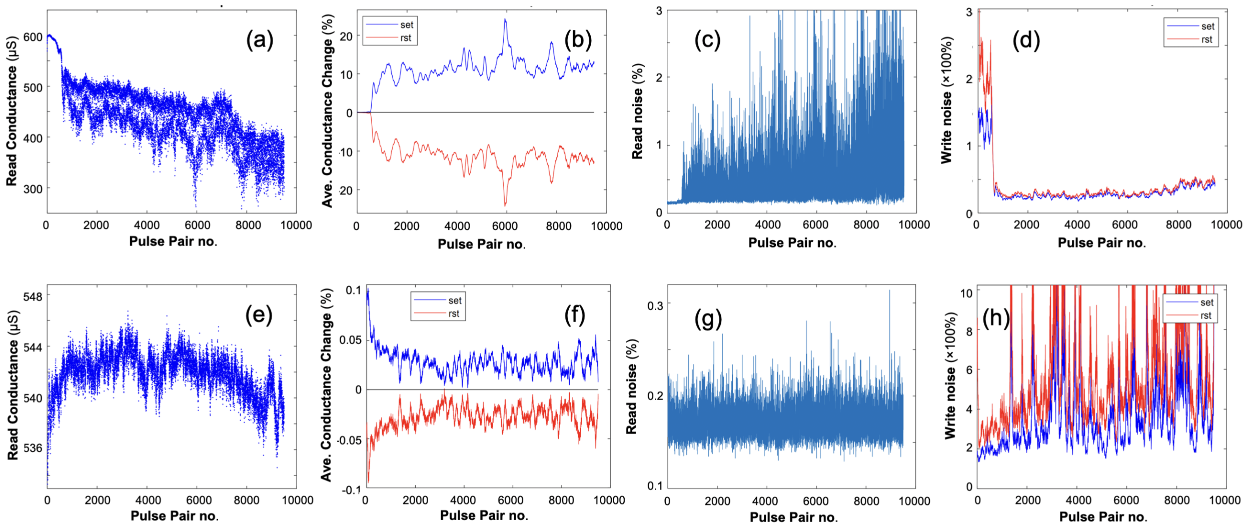

Figure S2: Read conductance, write modulation, RMS read noise and write noise (running average) for two neighboring RRAM devices (a-d) and (e-h) under identical pulsing conditions ($V_p = 1.4V$, $V_n = 1.7V$, $t_p = 10ns$, $P = 2ms$). Blue is for positive and red for negative programming pulses. Note the approach to symmetry in (b) and (f) (a, c). Note the large noise and write modulation in the 1st compared to the 2nd device. (b,c,d,f) are normalized to the running average of the conductance, and (d,h) are averaged and normalized to the running average of the absolute modulation.

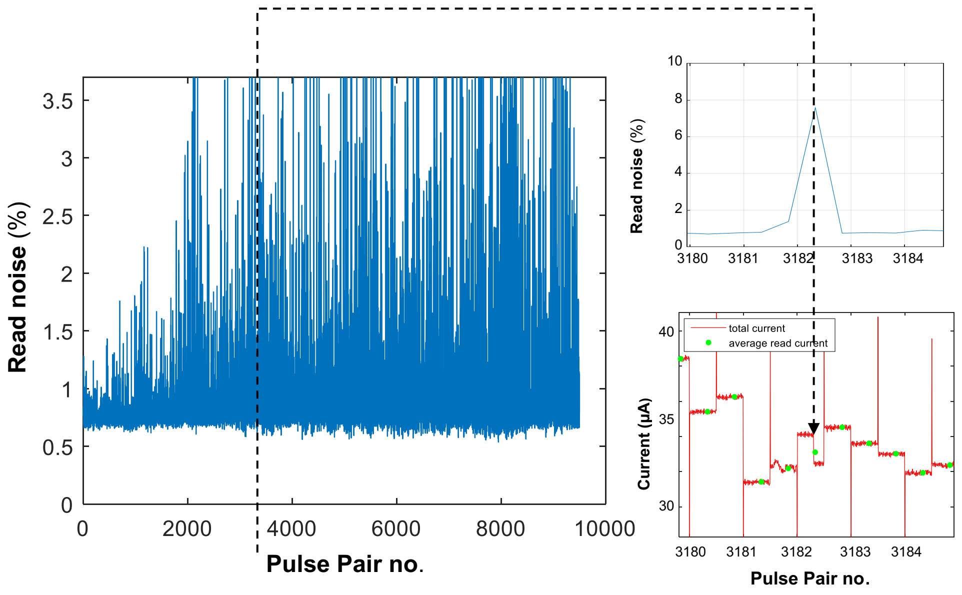

Figure S3: Detailed examination of the read noise, from the measurements in Fig. S2 (a-d), shows that the high noise peaks may be traced to RTN transitions. The spikes in the bottom right sub-figure are programming pulses. It is also seen that the read noise increases with number of programming pulse pairs.

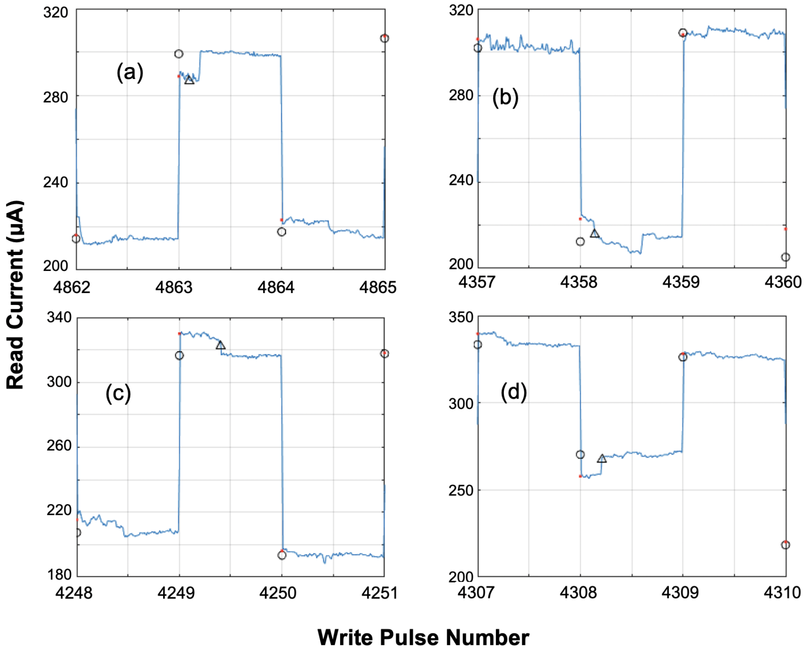

Figure S4: Four types of RTN transition, from the measurements in S2 (a-d), showing (a) completion of a set operation, (b) completion of a reset operation, then recovery, (c) recovery after a set and (d) after a reset operation. The small circles mark the average currents during the following $\frac{1}{2}$ period and the triangles mark the transitions detected with our detection method (see main text Fig. 1(d)).

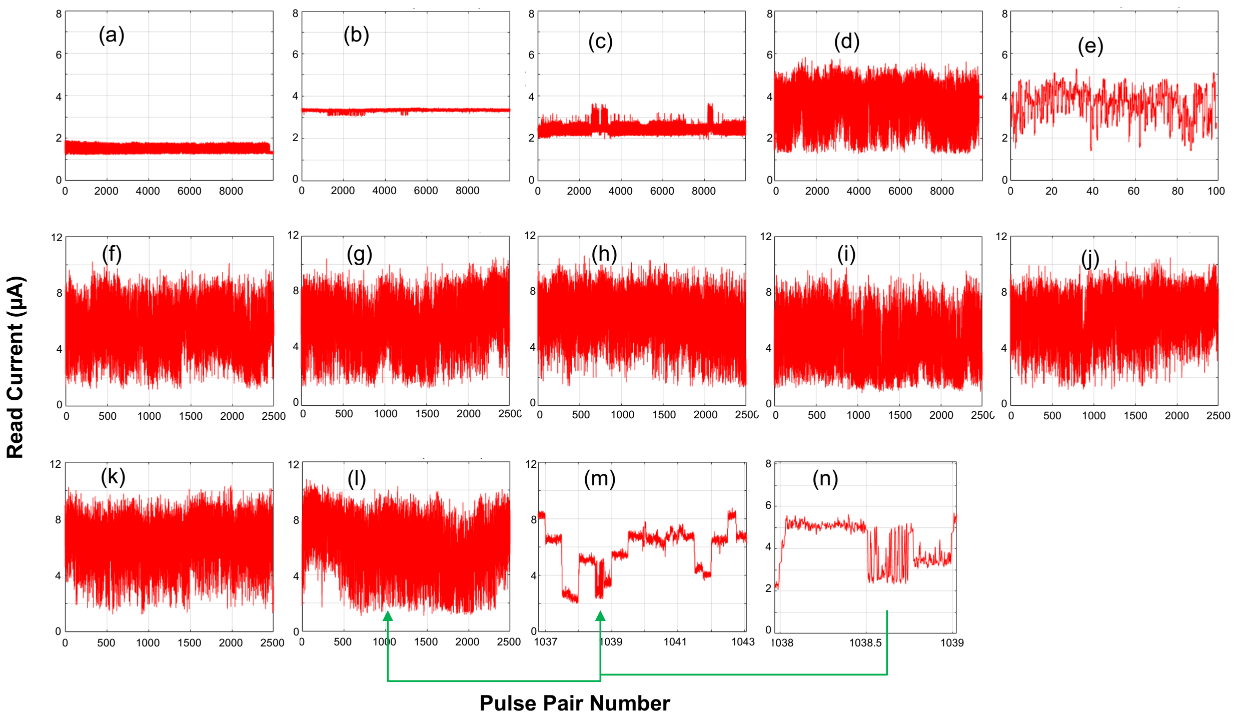

Figure S5: Sequence of up/down pulse experiments on the same sample, each 20s in duration, with a sampling period of 10 µs (a – e) and of 1µs (f - j). The panes (d and e) correspond to Fig. 1 (b and a) in the main text, and (f – l) match the repair voltage points in Fig. 3. The panes (m and n) show expanded views of (l) which include a burst of RTN. Pulse conditions are given in Table S1, for each pane. Numbers of RTN transitions with a threshold of 1mS for (a-e) and 3mS for (f - l) are also given in the table.

Table S1: Run Parameters for cases shown in Fig. S5. The letters refer to the panes in Fig. S5. V_h: repair pulse amplitude; V_p: positive programming pulse amplitude; V_n: negative programming pulse amplitude; t_p: pulse width (all pulses); trd: read interval (time are approximate accounting for transients) ; t_s: sampling interval; P: full period (two programming pulses + 2 read intervals); N_{RTN}: total number of RTN pulses detected in the 20s sweep.

pane*	cross-ref.	V_h (V)	V_p (V)	V_n (V)	t_p (µs)	trd (ms)	t_s (µs)	P (ms)	NRTN (×1000)
a	first	–	1.5	-1.5	1	∼1	10	2	0
b		–	0.125	0.75	1	∼1	10	2	0
c	Fig. 3	–	0.125	0.75	1	∼1	10	2	10.6
d	Fig. 2b	–	1.5	-1.5	1	∼1	10	2	5.5
e	Figs 2a, 4b,c	–	1.5	-1.5	1	∼100	10	200	2.7
f	Figs 4d, S8	-0.75	1.6	-1.6	1	∼2	1	8	32.9
g	"	-0.50	1.6	-1.6	1	∼2	1	8	31.8
h	"	-0.25	1.6	-1.6	1	∼2	1	8	34.0
i	"	0.10	1.6	-1.6	1	∼2	1	8	34.2
j	"	0.25	1.6	-1.6	1	∼2	1	8	32.8
k	"	0.50	1.6	-1.6	1	∼2	1	8	33.6
l	"	0.75	1.6	-1.6	1	∼2	1	8	33.3

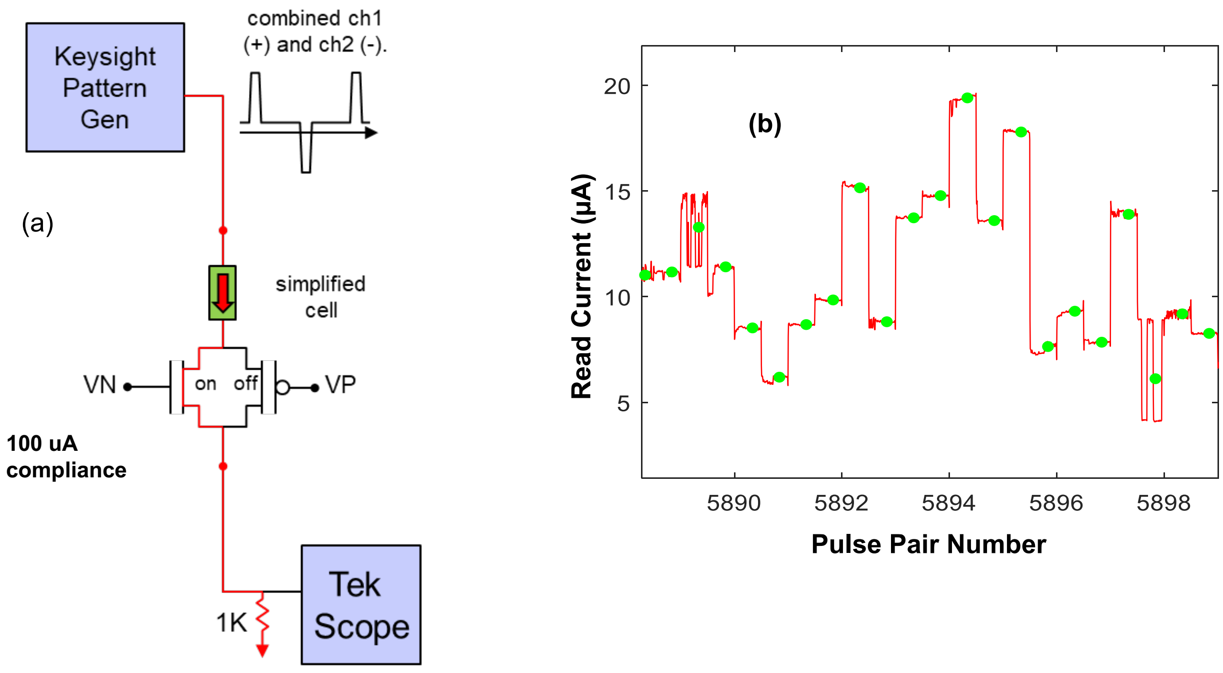

Figure S6: (a) Measurements using integrated complementary FETs in series with the RRAM device (2T1R). (b) Multiple RTN peaks being generated by a write operation and quenched by the next write operation. the pulse conditions were: V_p = 1.4V, V_n = 1.4V, t_p = 10ns, P = 0.2ms.

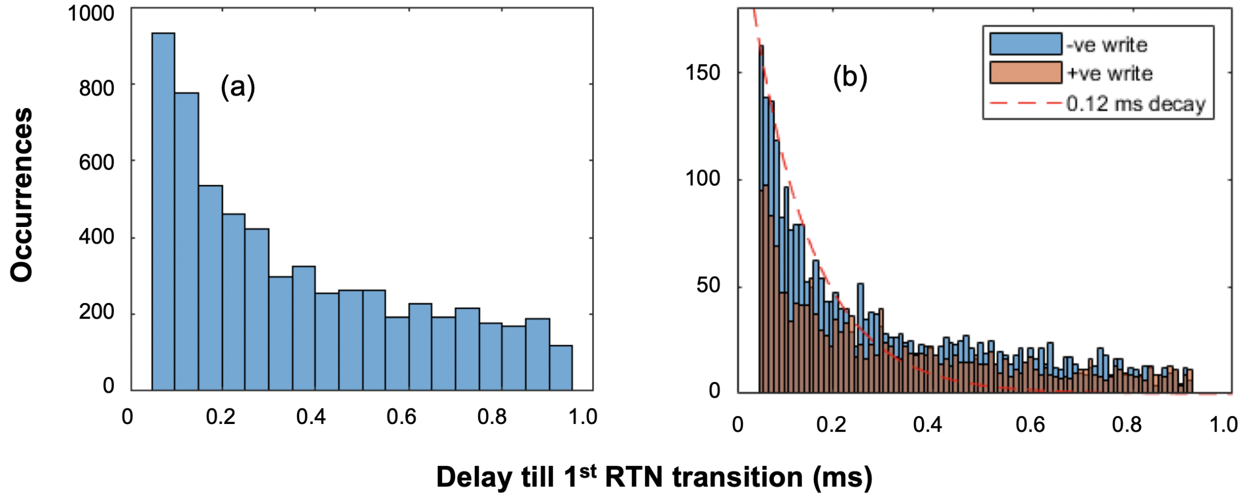

Figure S7: Statistics of the time delay between the write pulse and the first RTN transition. (a) for the sample in Fig. S2 (a-d), and (b) for the sample in S6 (d). The pulses were 1ms in length, so this plot covers intra-pulse transitions. The initial part of (b) is fitted to a decaying exponential (Poissonian statistics), although there is clearly a non-Poissonian tail, with a time constant of approximately 0.12 ms. Note that the devices in (a) & (b) originate from disparate fabrication runs, yet show similar statistical properties.

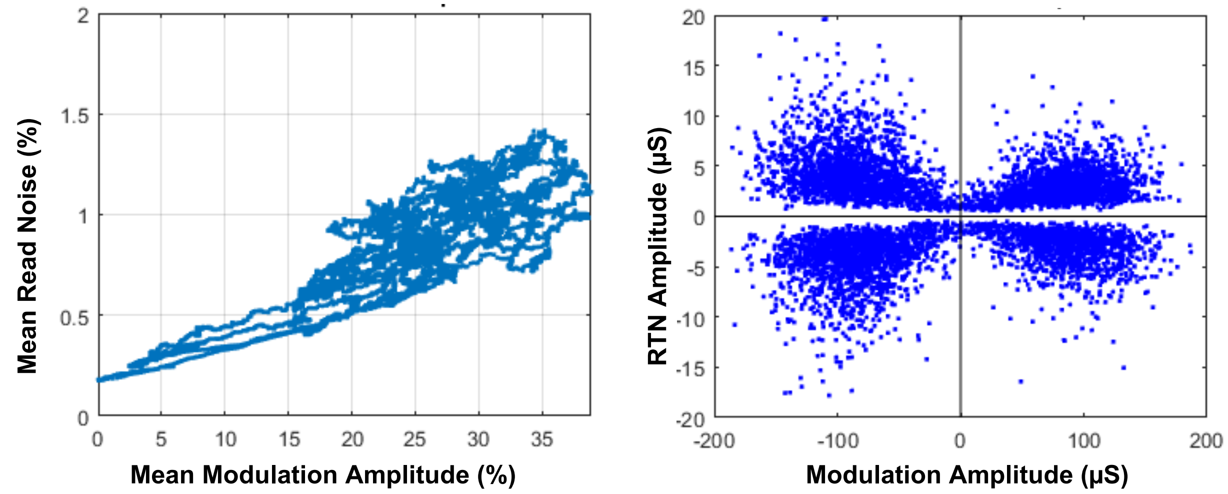

Figure S8: This sample shows strong correlation between average modulation amplitude and average amplitude of the associated RTN transitions. The blank strip at low RTN amplitude is an artifact of the selection process (6 sigma above background noise) to find valid transitions. The sample was from the same chip as in Fig. S2 and the pulse conditions were: $V_p = 1.4V$, $V_n = 2.1V$, $t_p = 10ns$, $P = 2ms$.

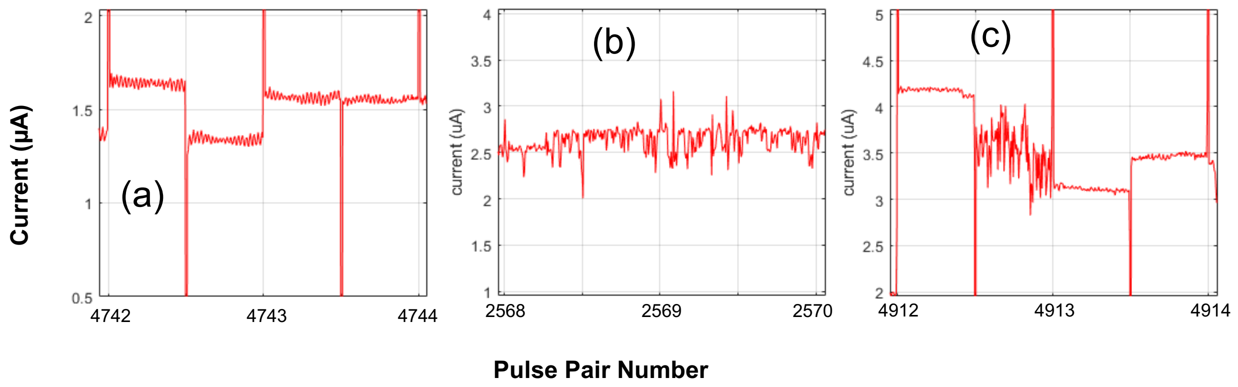

Figure S9: Phenomenology (corresponds to Fig. S5 (a, c, d)): (a) shows good modulation and no RTN. This is the first programming sequence on the freshly formed filament. (b) shows RTN but no modulation and (c) shows an extended burst of RTN bounded by write pulses. This is similar to the burst seen in Fig. 2 of the main text, but here the pulse period is 2ms rather than 200ms.

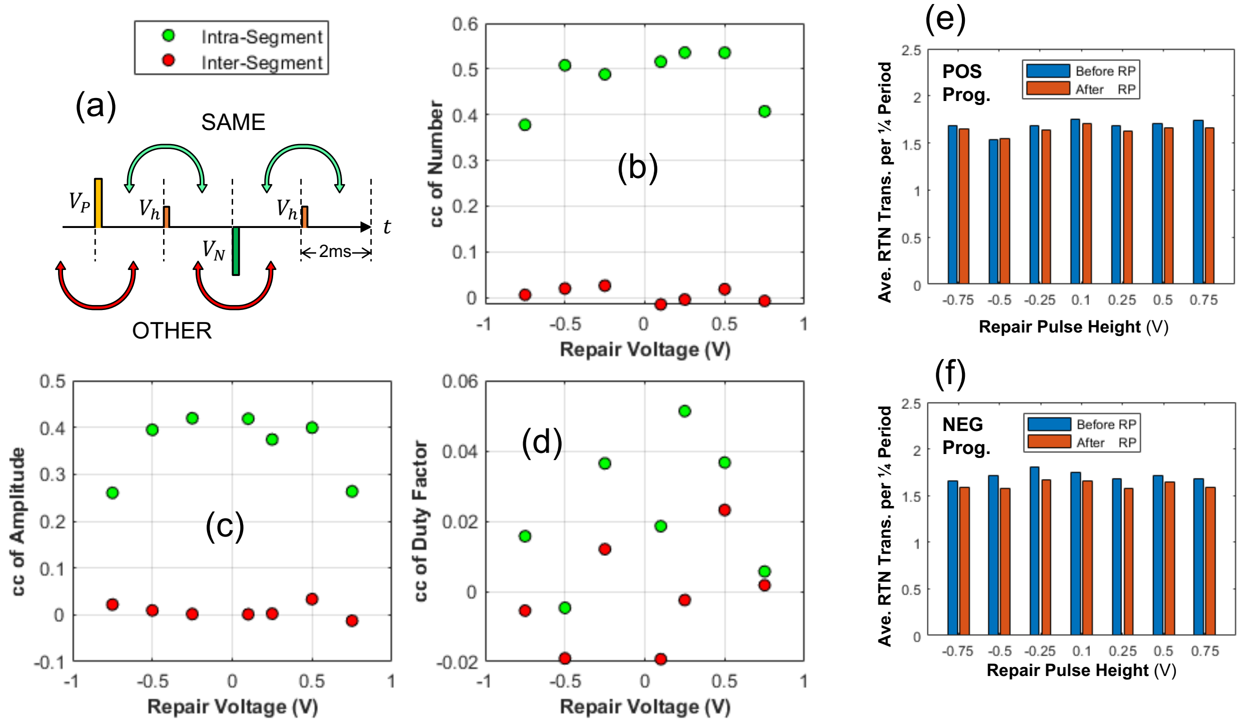

Figure S10: This is an expansion of Fig. 4d in the main text. Influence of insertion of a repair pulse, of amplitude Vh, between the two programming pulses (a). (b)-(e) Plots of correlation coefficient vs. Vh for the quantities: (b) number of RTN pulses, (c) amplitude of the RTN pulses, and (d) duty factor. Average number of transitions per $\frac{1}{2}$ programming segment before and after the repair pulse (e) after positive programming and (f) after negative programming pulses.

Supplementary Video 1, caption (see vacancy_oscillations_movie.mp4): Animation of the simulated atomic movements which lead to the observed bursts in Fig. 5b of the main manuscript.

Initially, we zoom in near the Ti/HfO_x interface, and highlight (with a yellow box) the location of the vacancy whose motion results in abrupt changes in current. The leftmost plot shows the atomic structure colored by defect type (red = displaced Oxygen, purple = Oxygen vacancy, grey = Ti, grey lines = HfO_x lattice atoms. The middle plot shows the same structure colored by the local potential, while in the rightmost each atom is colored by the local temperature profile. We then switch to a simplified view where the structure on the right omits the lattice atoms, and pictures only defect sites and the Ti contact atoms. The rightmost plot shows the current as in Fig. 5b, now magnified to the regime where sudden bursts are observed. The black dot tracks the exact value of current as a function of time during the structural re-arrangements occurring on the left. We omit showing the transitions between snapshot 301 to 540 for brevity.
